# Supplementary material for: Shedding a Light on Acyclovir Pharmacodynamics: A Retrospective Analysis on Pharmacokinetic/Pharmacodynamic Modelling of Acyclovir for the Treatment of Varicella Zoster Virus Infection in Immunocompromised Patients: A Pilot Study
Source: Pharmaceutics. 2022 Oct 27;14(11):2311. doi: 10.3390/pharmaceutics14112311 (PMC9695373; doi:10.3390/pharmaceutics14112311)
Supplement: Supplementary file 1 [file pharmaceutics-14-02311-s001.zip › pharmaceutics-1958403-supplementary.pdf]

pharmaceutics

MDPI

Supplementary Materials

Supplementary methods

VZV viral loads analysis

Varicella-zoster virus (VZV) viral loads were determined by quantitative real-time PCR. Nucleic acids were purified from 190µL of EDTA anti-coagulated plasma or serum by either MagNA Pure 96 Total Nucleic Acid Isolation Kit [MP96] (patient 1, Roche Diagnostics, Mannheim, Germany) or NucliSENS easyMAG/eMAG [EM] (patients 2-4, bioMérieux, Marcy l'Etoile, France), according to manufacturer's instructions. Nucleic acids were eluted in 100 (MP96) or 110 µL [EM] of elution buffer. Quantitative real-time PCR was conducted using the TaqMan Universal Master Mix (Thermo Fisher Scientific, Waltham, MA USA) and the primer/probe set described by Hawrami et al. on the 7500 Real-Time PCR System (Thermo Fisher Scientific) [18]. Reaction mixtures consisted of 1x TaqMan Universal Master Mix (Thermo Fisher Scientific), 5 µg Bovine Serum Albumin (Roche Diagnostics), Forward primer at 600 nM, Reverse primer at 300 nM, FAM-TAMRA dual labelled TaqMan probe at 100 nM and 20 µL of template nucleic acid. Cycling conditions: 2 min at 50°C, then 10 min at 95°C followed by 42 repeats of 15 seconds at 95°C, and 1 min at 60°C.

PK model

A population pharmacokinetic (PK) model was built in Pmetrics. A one-compartment model of the Laboratory of Applied Pharmacokinetics and Bioinformatics was used as the PK starting model. Elimination rate constant (K_e) in h^{-1}, volume of distribution (V) in L, and absorption rate constant (k_a) in h^{-1} were used as PK parameters.

A one- and two-compartment model were tested. Correlations between PK parameters and covariates were evaluated and tested in the model: serum creatinine, weight, gender, age, height, body mass index (BMI), lean body mass (LBM), body surface area (BSA), and estimated glomerular filtration rate (eGFR). Margins of the PK parameters were adjusted, bioavailability (FA) was added and an additive and proportional error were tested in the model.

PK model code

```
#Pri
Ke, 0.001, 1
V, 1, 70
Ka, 0.001, 1
FA1, 0.001, 1

#F
FA(1) = FA1

#Out
Y(1) = X(2)/V

#Err
L=0.1
0.1,0.1,0,0

#cov
SER_CREAT
WEIGHT
GENDER
AGE
HEIGHT
BMI
LBM
BSA
eGFR
```

PK/PD model code

```
#PRI
Kkmax,0.001,0.5
Hk, 0.1, 30
IC,0.01,500000
EC50, 0.001, 30

#COV
eGFR
Ke1
V1
Ka1
FA1

#F
FA(1) = FA1

#INIT
X(3)=IC

#DIF
XP(1) = -Ka1*X(1)
XP(2) = Ka1*X(1) - Ke*X(2)
XP(3) = -Kkmax * ((X(2)/V1)**Hk / ((X(2)/V1)**Hk + EC50*Hk)) * X(3)

#OUT
Y(1)=DLOG10(X(3))

#Err
G=5
0.05,0.1,0,0
```

Table S1. Patient characteristics for population pharmacokinetic modelling.

Patient characteristics	Median	Range
Number of patients	43	
Age (years)	57.0	2-81
Weight (kg)	80.0	14-140
Length (cm)	175.0	87-192
BMI (kg/m2)	25.3	14-46
Serum creatinine (µmol/L)	87.0	13-279
eGFR (mL/min/1.73m2)	76.0	26-255
Gender (male/female)	53% / 47%	
Administration: ACV IV / VCV / both	14 / 17 / 12	

BMI: body mass index; eGFR: estimated glomerular filtration rate; ACV IV: acyclovir intravenous; VCV: valacyclovir.

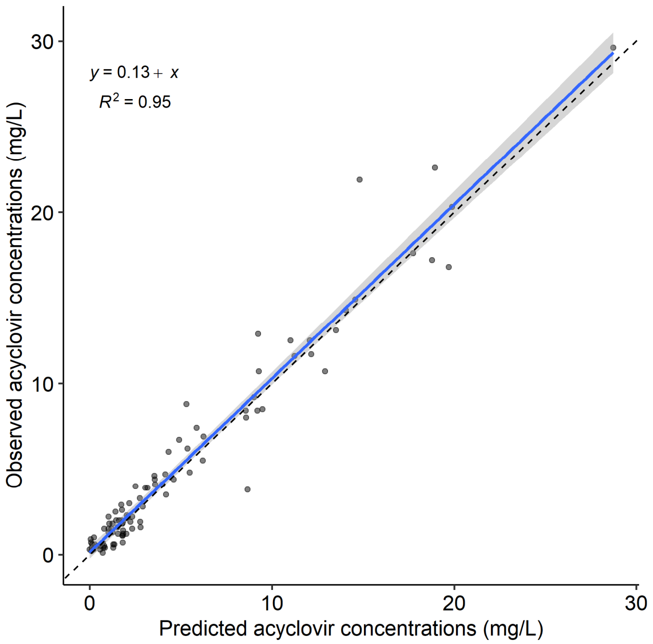

Figure S1. Individual posterior predictions goodness-of-fit plot of final pharmacokinetic model (N = 43). The solid line represents the linear regression of the observed and predicted acyclovir concentrations, the dashed line represents a reference line for y = x and the black dots display the observed acyclovir concentrations.
